# Matrix‐Dependent Sweet–Sour Psychophysics: gLMS and Time‐Intensity of Sugar Alcohol‐Acid Mixture in Liquids and Tablets

**DOI:** 10.1002/fsn3.71610

**Published:** 2026-03-11

**Authors:** Chenchen Liu, Yuting Wang, Jinxia Bai, Yuezhong Mao, Shiyi Tian

**Affiliations:** ^1^ Zhejiang Institute for Food and Drug Control Zhejiang China; ^2^ School of Food Science and Biotechnology Zhejiang Gongshang University Zhejiang China; ^3^ Zhejiang Sochi NutriOne Technology Co. Ltd. Zhejiang China; ^4^ China‐UK Joint Research Laboratory of Eating Behaviour and Appetite Zhejiang Gongshang University Zhejiang China

**Keywords:** acidulant, gLMS, sugar alcohol, sweet–sour interaction, time‐intensity

## Abstract

Precision control of sweet–sour interactions is pivotal for reduced‐sugar design across formats. This work quantified psychophysical functions for xylitol, erythritol, and sorbitol with citric or malic acid in liquids and tablet candies using gLMS concentration‐intensity and time‐intensity methods. In liquids, subthreshold ratings clustered near barely detectable, while suprathreshold relations followed Stevens' power law; exponents declined at extreme levels, indicating saturation. Tablets preserved power‐law scaling yet exhibited > 79% higher acid exponents than liquids, producing steeper sourness growth; in erythritol and sorbitol, malic exceeded citric by > 21%, whereas exponents were ~1 and comparable in xylitol. TI results showed bidirectional suppression: acids lowered sweetness (*I*_max_, AUC), with xylitol displaying compressed onset‐decay‐persistence; sugar alcohols attenuated sourness, with malic less inhibited than citric. These parameters enable predictable, matrix‐specific tuning to preserve sweetness, prevent sourness spikes, and support evidence‐based formulation for consumer acceptance, regulatory sugar targets, shelf‐life stability, and scalability.

## Graphical abstract

Tablets showed > 79% steeper sourness growth than liquids by power law. Malic acid yielded steeper sourness growth than citric acid in most matrices and xylitol matrix yielded balanced perception with near‐unity sourness exponents. Time‐intensity parameters provide actionable targets for controlling sweetness‐sourness balance.

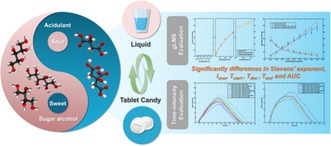

## 1 Introduction

Sugar alcohols—such as xylitol, erythritol, and sorbitol—were widely used in beverages, confectionery, chewing gum, and in oral‐care and pharmaceutical solid formulations because they provided bulk while enabling sugar reduction (Junge et al. 2022). Acidulants—such as citric and tartaric acids—imparted sourness, adjusted pH, enhanced freshness, and improved product stability (Mao et al. 2021). In industrial formulations, sweeteners and acidulants commonly co‐occurred, and their interactions shaped overall flavor intensity and temporal profiles, thereby influencing consumer perception and acceptance (Mao et al. 2022). Nevertheless, quantitative, matrix‐specific rules for managing the sweet–sour balance remained limited; establishing robust psychophysical parameters to predict these interactions was critical for designing reduced‐sugar products that consistently met sensory targets (Mao et al. 2018).

From a theoretical perspective, the perception of mixed tastes was often non‐additive rather than a simple linear summation (Veldhuizen et al. 2018). Suppression and synergy represented common departures: at moderate to high concentrations, sourness typically suppressed perceived sweetness, whereas at lower acid levels a mild sour sensation enhanced freshness and increased the salience of sweetness, producing a more pleasant overall flavor than linear addition would predict (Hallowell et al. 2016; Low et al. 2017). In addition, when sourness co‐occurred with congruent sensory cues, including citrus aroma and oral texture, sour–sweet mixtures were perceived as more vivid and complex, reflecting cross‐modal integration that could further strengthen the overall flavor impression (Tepper et al. 2025). These phenomena were contingent on stimulus type, concentration range, and carrier form (e.g., liquid or solid), as well as by inter‐individual variability. Consequently, empirical work needed to track intensity changes within well‐defined concentration windows to characterize interaction functions and to inform application‐relevant formulation decisions.

Within this measurement context, the general labeled magnitude scale (gLMS) served as an important tool for comparing intensities across individuals and stimuli (Kan et al. 2025). By anchoring ratings to quasi ratio verbal descriptors, it yielded more comparable data and reduced bias related to individual or cultural differences, and it was consequently used widely to study sweeteners, acidulants, and their interactions. Comparative studies with the generalized visual analog scale (gVAS) indicated that gLMS provided greater discriminative power, repeatability, and stability (Hayes et al. 2013). When combined with power law modeling, the approach allowed researchers to extract parameters that compactly represented the nonlinear relation between stimulus and perceived intensity, thereby supplying a practical basis for psychophysical analysis and for comparing matrices and delivery formats (Mennella et al. 2015).

In the domain of dynamic measurement, time‐intensity (TI) analysis quantified onset, peak, decay, and area under the curve (AUC), thereby describing the time course of flavor perception during consumption (Bian et al. 2023). Multi‐attribute temporal methods (e.g., TDS and related visualization/analysis tools) captured how multiple attributes co‐evolved over time and were suited to complex and multi‐dimensional flavor systems (Batali et al. 2020). In solid or tablet matrices, where release conditions during use could differ from those in liquids, the value of these methods became more apparent. Accordingly, combining static gLMS with dynamic TI or TDS approaches allowed researchers to address both how strong a given dose was and how that intensity changed over time (Mao et al. 2025).

Although the preceding theoretical and measurement framework provided a foundation, important gaps remained in the available evidence. First, most studies focused on sucrose with a limited set of acidulants in aqueous models, and findings largely emphasized suppression of sweetness by sourness; this effect was reported across populations (Pelletier et al. 2004). Second, the implementation of scaling methods, including the choice and training for gLMS versus gVAS and the conditions for between population comparisons, was not harmonized, which affected intensity estimates and hindered cross‐study comparability (Bartoshuk et al. 2003). Third, with respect to dynamic methods, studies compared the applicability of TI and TDS and introduced TCATA to describe the temporal evolution of multiple attributes (Castura et al. 2016; Pineau et al. 2009), yet systematic evidence on sugar alcohols such as xylitol, erythritol, and sorbitol across wide concentration ranges, as well as on their time course interaction with sourness, remained limited. Moreover, matrix diversity was narrow, and comparable data for solid or tablet formats were scarce, making it difficult to determine whether matrix changes altered the observed interactions. These gaps underscored the need for harmonized, matrix‐matched measurements that spanned subthreshold to suprathreshold ranges and captured both static and dynamic responses.

Recent advances in dynamic methods offered a practical way to address these limitations. Time intensity quantified key temporal indices such as onset, peak, decay, and area under the curve, and temporal dominance of sensations and temporal check all that apply tracked dominant and concurrent attributes over time. Studies on the temporal precision and resolution of these approaches supported the acquisition of more comparable dynamic outcomes within predefined low, medium, and high intensity ranges (Visalli et al. 2024). Together with static gLMS and power law fits, this framework enabled systematic and matrix sensitive comparisons of sugar alcohol by acidulant mixtures across liquid and tablet matrices under aligned scale implementation, harmonized concentration windows, and consistent statistical procedures.

**FIGURE 1:**
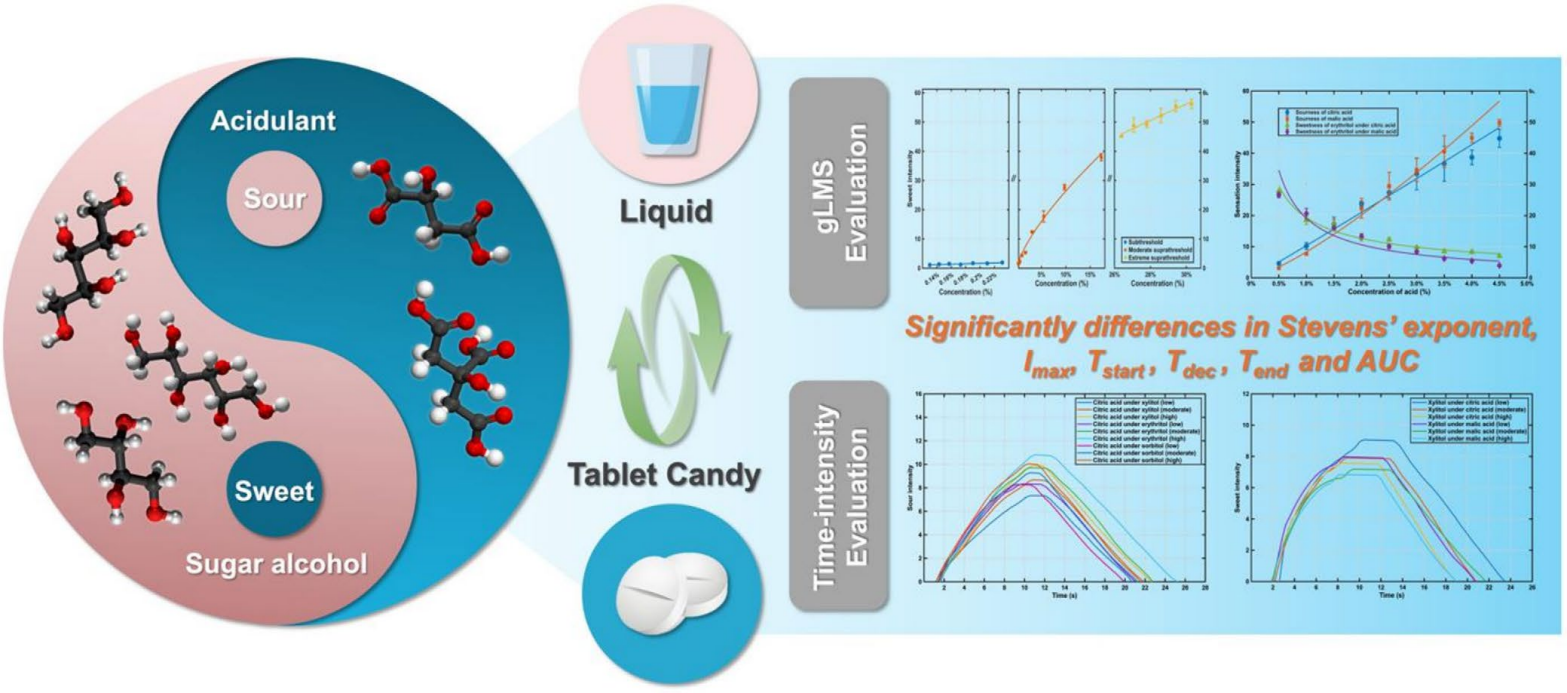
Schematic diagram of this research.

Guided by the above gaps and methodological considerations, this study examined three sugar alcohols and two acidulants and applied gLMS and TI in parallel across two matrices, liquid and tablet. First, gLMS was used to construct concentration response functions spanning very low to very high levels. Second, in liquids, gLMS and TI jointly characterized the static intensity and the temporal trajectory of sweet and sour interactions. Third, the same measurement framework was extended to tablet candy to compare perceptual outcomes between the two matrices under identical procedures, as shown in Figure 1. Together, the findings established reproducible, matrix sensitive psychophysical parameters and reference datasets that enabled predictive tuning of sweet and sour balance, advanced mechanistic understanding across delivery formats, and supported the rational design of reduced sugar products with consistent consumer acceptance.

## 2 Materials and Methods

### 2.1 Materials and Chemicals

Xylitol, erythritol, and sorbitol were purchased from Zhejiang Huakang Pharmaceutical Co. Ltd. Citric acid, malic acid, and magnesium stearate were purchased from Shanghai Macklin Biochemical Technology Co. Ltd. Ultra‐pure water was prepared using Milli‐Q water, resistivity of 18.2 MΩ·cm by using a Millipore system.

### 2.2 Sample Preparation

#### 2.2.1 Single‐Taste Liquid Samples for gLMS

The absolute threshold and terminal threshold values of xylitol, erythritol, sorbitol, malic acid and citric acid were determined by triangle test via our previous study (Mao et al. 2019). Here, the absolute threshold was defined as the lowest concentration that panelists could reliably detect relative to blank, and the terminal threshold as the highest concentration that panelists could still tolerate and evaluate reliably. To span the perceptual range while maintaining appropriate resolution, concentrations were designed a priori into three segments. The subthreshold segment centered on the absolute threshold and included three concentrations below and three above it at 1/24 log10 spacing (factor 1.101). The moderate suprathreshold segment started at the absolute threshold and extended upward with eight concentrations at 1/4 log10 spacing (factor 1.778) for Stevens power law fitting. The extreme suprathreshold segment targeted the high intensity region approaching the terminal threshold, using the terminal threshold as a reference and applying finer 1/36 log10 spacing (factor 1.066) to capture near ceiling behavior.

#### 2.2.2 Sweet–Sour Mixture Liquid Samples for gLMS

The low, medium, and high concentrations of sweet and sour backgrounds were determined through panel evaluation and discussion. Specifically, xylitol was set at 22, 55, and 110 g/L; erythritol at 32, 65, and 150 g/L; sorbitol at 34, 90, and 170 g/L; citric acid at 0.25, 0.45, and 0.80 g/L; and malic acid at 0.30, 0.55, and 0.95 g/L. Subsequently, the low, medium, and high background concentrations were incorporated into the moderate suprathreshold segments of xylitol, erythritol, sorbitol, citric acid, and malic acid to prepare sweet–sour mixture samples for gLMS evaluation.

#### 2.2.3 Sweet–Sour Mixture Tablet Candy Samples for gLMS

The sweet–sour mixture tablet candy samples were prepared using sugar alcohol, acidulant, and magnesium stearate. To meet the manufacturing requirements of the tablet candy, the content of sugar alcohol was fixed at 95%, while the content of acidulant varied from 0.5% to 4.5%, with a corresponding adjustment in the content of magnesium stearate from 4.5% to 0.5%. Detailed sample information was provided in Table S1.

#### 2.2.4 Sweet–Sour Mixture Liquid Samples for TI

Based on the previous studies and pre‐experimental results, the low, moderate, and high sourness background levels were defined using citric acid at 0.25, 0.45, and 0.80 g/L, and malic acid at 0.30, 0.55, and 0.95 g/L. The evaluation concentrations for xylitol, erythritol, and sorbitol were 55, 65, and 90 g/L, respectively.

Accordingly, the low, moderate, and high sweetness background levels for xylitol, erythritol, and sorbitol were 22, 32, 34, 55, 65, 90, 110, 150, and 170 g/L. The evaluation concentrations for citric acid and malic acid were 0.45 and 0.55 g/L, respectively.

#### 2.2.5 Sweet–Sour Mixture Tablet Candy Samples for TI

Consistent with the samples prepared for gLMS evaluation, the tablet candy formulations were likewise composed of sugar alcohol, acidulants, and magnesium stearate. The sugar alcohol content was fixed at 95%, while the concentration of acidulants was determined based on a Just‐About‐Right (JAR) test to achieve three distinct taste profiles: slightly sour, balanced sweet–sour, and predominantly sweet. Detailed sample information is provided in Table S2.

### 2.3 Sensory Panel

The sensory panel comprised 30 selected assessors (15 males and 15 females, aged 20–35 years), recruited in accordance with the Chinese national standard GB/T 16291.1–2012 Sensory analysis—General guidance for the selection, training, and monitoring of assessors—Part 1: Selected assessors and ASTM E1909‐13 (2017). Each assessor was informed of the study objectives and completed a two‐week training program, conducted once daily. The training included the following components:

1. Sensory evaluation terminology: Assessors received instruction on terms such as intensity, dynamics, and liking, to establish a consistent descriptive framework.
2. Basic taste discrimination: Assessors were required to correctly identify and differentiate basic taste sensations.
3. Methodological training: gLMS and TI procedures were introduced and practiced
4. Accuracy in taste intensity evaluation: The relative error in rating the intensity of the same concentration was required to remain below 10%.

In addition, assessors were instructed to refrain from smoking or eating for at least 30 min before each evaluation session to minimize potential interferences.

The whole study was approved by the Ethics Committee of Zhejiang Gongshang University (No. 23134703). All participants were asked to provide informed consent prior to participation, with explicit information about data confidentiality and voluntary withdrawal rights.

### 2.4 Sensory Evaluation

#### 2.4.1 gLMS Evaluation

The gLMS evaluation was performed on our self‐established software. The gLMS was displayed vertically on a tablet screen, and participants responded by sliding the cursor to the appropriate position on the scale. The gLMS ranged from 0 to 100, with intensity descriptors anchored at specific values: 0 (“no sensation”), 1.4 (“barely detectable”), 6 (“weak”), 17 (“moderate”), 34.7 (“strong”), 52.5 (“very strong”), and 100 (“strongest imaginable of any kind”).

The liquid samples were poured into 50 mL plastic cups, with each cup containing a 10 mL aliquot. The presentation order of the samples was randomized within the concentration range. Participants were instructed to take the entire sample solution into their mouths and then score its intensity on the software. Before and after evaluating each sample, participants were required to rinse their mouths thoroughly, with a rest interval of 10 min between samples.

For the tablet candy samples, participants were instructed to chew using their molars, with the number of chews controlled to ten. They then assessed the taste intensity released at 10 s and recorded their ratings using the provided software.

#### 2.4.2 TI Evaluation

The TI evaluation was also performed on our self‐established software (Mao et al. 2025). Upon initiation of the test, participants consumed the liquid sample in one sip (or chewed the tablet candy sample) to ensure full oral exposure. During the test, they continuously perceived changes in taste intensity and adjusted the cursor position in the software in real‐time according to the perceived intensity until the taste sensation fully dissipated. The time intensity signal was recorded by the software at 0.01 s intervals and saved automatically. The endpoint “complete disappearance of taste” was participant determined.

## 3 Results and Discussion

### 3.1 Concentration‐Intensity Relationships of Sugar Alcohols and Acidulants

**FIGURE 2:**
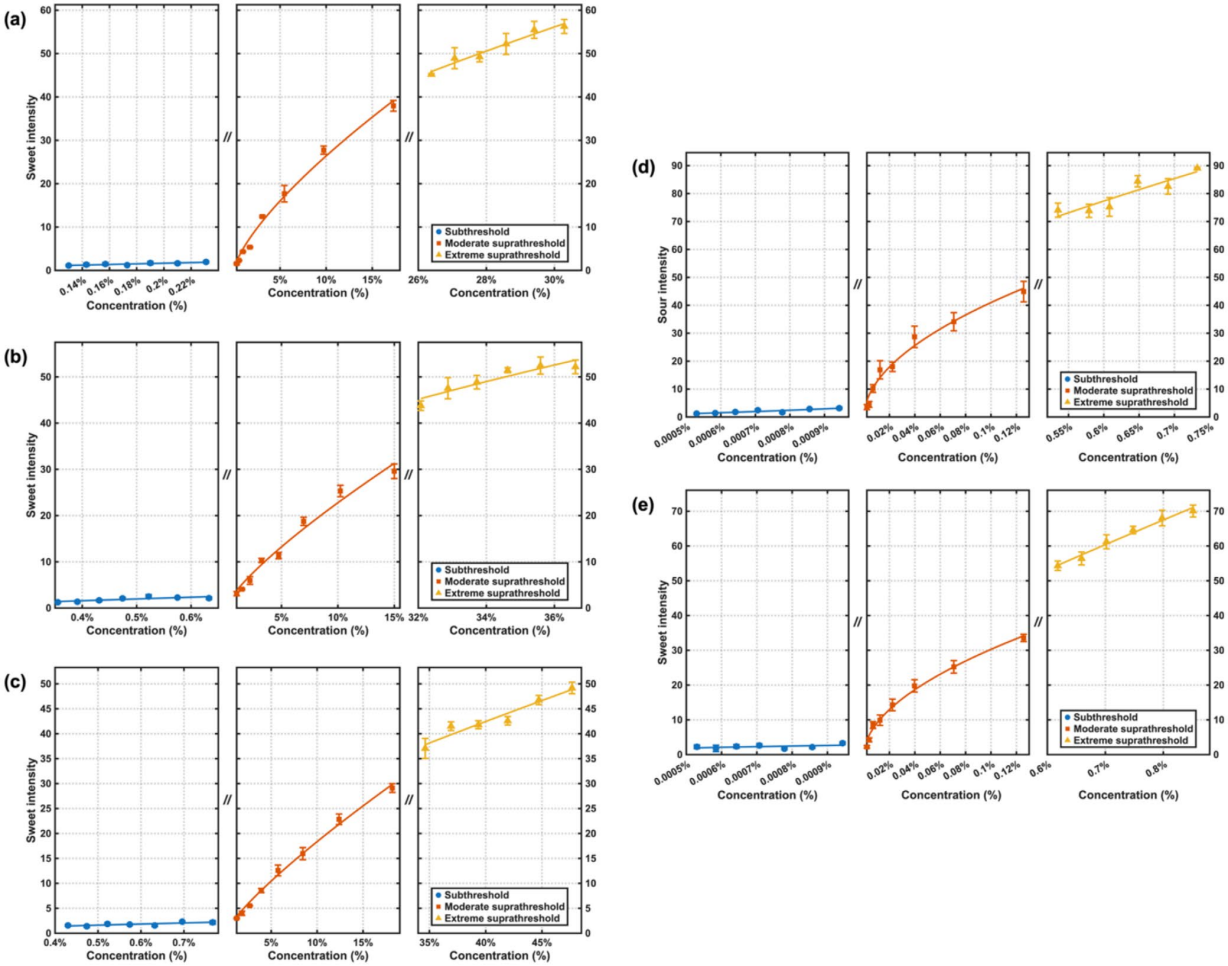
Concentration‐intensity curves of single sugar alcohols and acidulants liquid samples: (a) Sweetness of xylitol; (b) sweetness of erythritol; (c) sweetness of sorbitol; (d) sourness of citric acid; (e) sourness of malic acid.

The concentration‐perception intensity curves of sugar alcohols (xylitol, erythritol, sorbitol) and acidulants (citric acid, malic acid) liquid samples were obtained by plotting analyte concentration on the *X*‐axis against panel gLMS ratings of perceived sweetness or sourness on the *Y*‐axis, as shown in Figure 2a–e. To resolve behavior across the perceptual range, each function was segmented a priori into three regions defined by psychophysical anchors: a subthreshold region, a moderate suprathreshold region, and an extreme suprathreshold region.

As indicated in Figure 2, all five compounds exhibited a consistent trend across these perceptual ranges. In the subthreshold region, the perceived intensity fluctuated slightly, with ratings predominantly clustering between 1 and 2 on the gLMS, corresponding to a “barely detectable” level. This clustering indicated that panelists did not reliably discern intensity differences arising from concentration variation within this window, even when nominal concentration steps were present.

In both the moderate suprathreshold and extreme suprathreshold ranges, the perceived intensities of all five components increased with rising concentration. The maximum perceived sweetness of xylitol and erythritol ranged between 50 and 60, whereas that of sorbitol was approximately 50. In contrast, the maximum perceived acidity of citric acid and malic acid exceeded 70, suggesting that panelists exhibited a lower tolerance threshold for sweetness compared to acidity. At the same time, in the moderate suprathreshold and extreme suprathreshold region, all five compounds' concentration and perception intensity exhibited a power function relationship, which fitted the Stevens' power law. For each compound and region, the fitted equations and the associated indices are reported in Table 1.

**TABLE 1:** Fitted equation of concentration‐perception intensity curves for sugar alcohols and acidulants.

Substances	Moderate suprathreshold region		Extreme suprathreshold region	
	Fitted equation	Correlation coefficient (*R* ^2^)	Fitted equation	Correlation coefficient (*R* ^2^)
Xylitol	*Y* = 5.04*X* ^0.719^	0.989	*Y* = 5.05*X* ^0.677^	0.962
Erythritol	*Y* = 3.75*X* ^0.784^	0.978	*Y* = 7.48*X* ^0.528^	0.873
Sorbitol	*Y* = 2.84*X* ^0.811^	0.995	*Y* = 2.20*X* ^0.802^	0.934
Citric acid	*Y* = 133.93*X* ^0.514^	0.976	*Y* = 107.3*X* ^0.641^	0.846
Malic acid	*Y* = 102.06*X* ^0.528^	0.990	*Y* = 81.24*X* ^0.831^	0.982

The fitted formula and detailed indexes were listed in Table 1. Notably, in the extreme suprathreshold region, the sugar alcohol's fitted power‐law exponents were smaller than moderate suprathreshold, indicating that perceived intensity grows more slowly with additional concentration and tends toward an asymptote. This systematic reduction in fitted exponents signaled a slower rate of perceptual growth with additional concentration at the highest tested levels and an approach toward an asymptote in the psychophysical function. In contrast, the power‐law exponents of citric acid and malic acid are higher in the extreme suprathreshold region than in the moderate suprathreshold region. This suggested that panelists' perception of acidity continues to increase even as concentrations approach the upper threshold limit, without a marked reduction in sensitivity. This observation further provided an explanation for the higher maximum perceived acidity relative to maximum perceived sweetness.

The observed patterns accord with established principles in taste psychophysics and help situate the present findings within the broader literature. On the gLMS, intensities around 1 to 2 correspond to the “barely detectable” anchor, and prior work has shown that near‐threshold changes in concentration are not reliably discriminated in this range; this has been documented for multiple sweeteners, including the sugar alcohol erythritol when thresholds and suprathreshold ratings were considered together (Low et al. 2017). The present subthreshold clustering therefore reflected a canonical near‐threshold insensitivity rather than a stimulus‐specific anomaly. At suprathreshold levels, concentration‐intensity relations for basic tastes are typically captured by power functions, and classic experiments spanning a broad set of acids reported sourness scaling with a mean exponent close to 0.85 (Moskowitz 1971).

The systematic reduction in fitted power‐law exponents for sugar alcohols in the extreme suprathreshold region invites a mechanistic interpretation consistent with the saturation of taste transduction. As concentrations increase and approach levels that nearly occupy available sweet taste receptor sites, the resulting neural signals from taste afferents drive toward their maximal response rates. Under this saturation scenario, incremental increases in concentration yield progressively smaller perceptual gains, a phenomenon reflected in the smaller exponents and the approach of the psychophysical function toward an asymptote.

In stark contrast, the observed increase in power‐law exponents for citric and malic acid in the extreme suprathreshold region suggests a fundamentally different perceptual behavior for sourness. This pattern indicates that the perception of acidity does not plateau as readily as sweetness but continues to grow robustly even at high concentrations. This divergence provided a compelling explanation for the higher maximum perceived acidity ratings (> 70 on the gLMS) compared to those for sweetness (50–60). The underlying mechanism may involve differences in receptor physiology; while sweet perception primarily involves G‐protein coupled receptors susceptible to saturation, sour taste is transduced via proton‐sensing ion channels, which may exhibit a wider dynamic range or less pronounced adaptation/saturation at high proton concentrations. This continuous growth in perceived intensity, uncompromised by saturation effects, likely contributes to the panelists' apparent lower tolerance for high levels of acidity, as the sensation remains salient and continues to intensify markedly across the concentration range.

### 3.2 Sweetness Responses of Sugar Alcohols Under Acidulants Backgrounds

**FIGURE 3:**
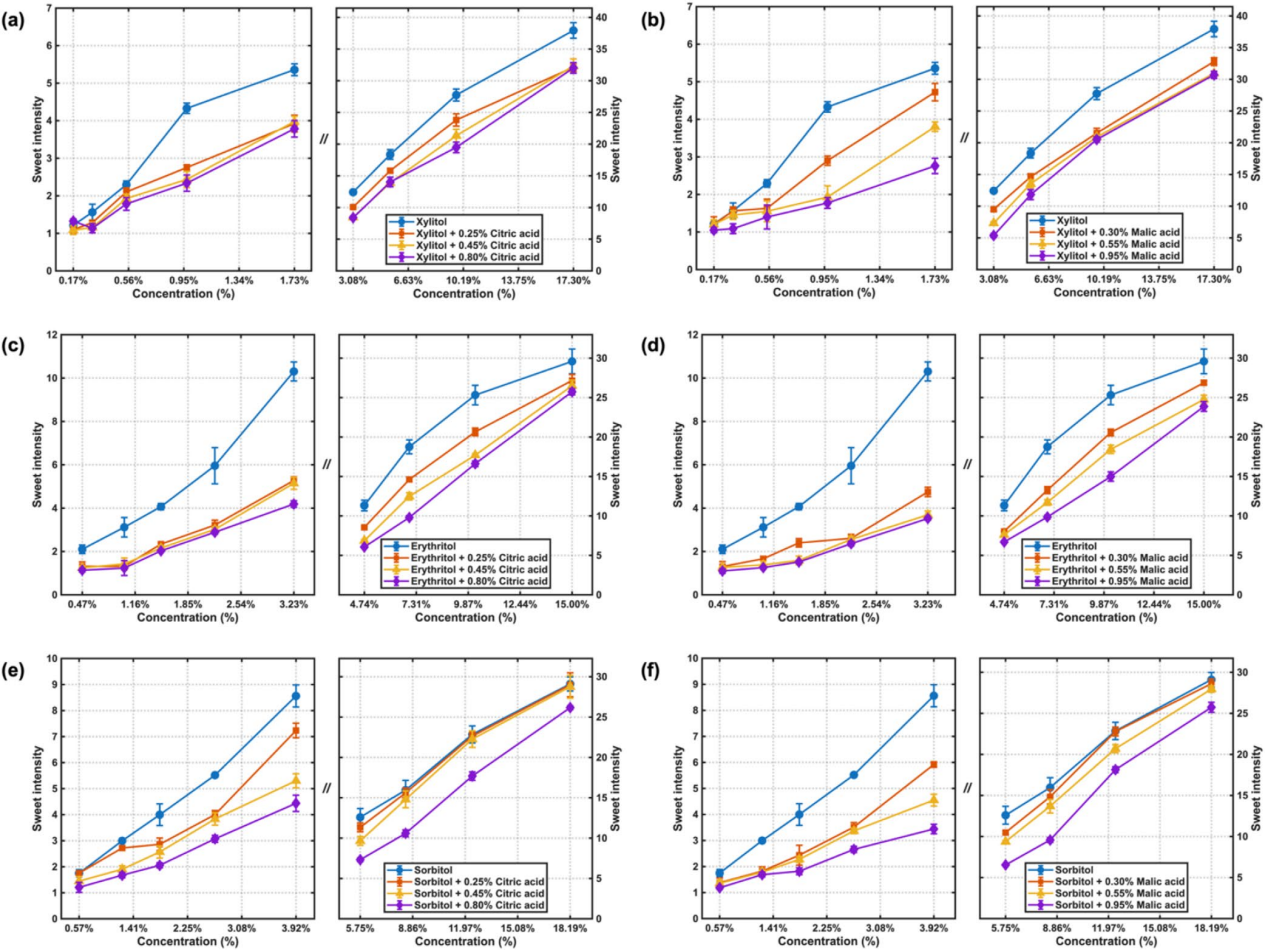
Concentration‐intensity curves of sugar alcohols in liquid acidulants samples: (a) Sweetness of xylitol under citric acid; (b) sweetness of xylitol under malic acid; (c) sweetness of erythritol under citric acid; (d) sweetness of erythritol under malic acid; (e) sweetness of sorbitol under citric acid; (f) sweetness of sorbitol under malic acid.

The perceived sweetness intensities of three sugar alcohols under varying backgrounds of two acidulants, as measured by the gLMS, are summarized in Figure 3. Specifically, Figure 3a–d depict the psychophysical functions for xylitol and erythritol across low, moderate, and high concentration levels of citric acid and malic acid, respectively. The corresponding power‐law fitting parameters and statistical indices for these relationships are detailed in Table 2.

**TABLE 2:** Fitted equation of interactive response curves of sugar alcohols' sweet intensity under acidulant background.

Substances	Fitted equation	Correlation coefficient (*R* ^2^)	Substances	Fitted equation	Correlation coefficient (*R* ^2^)
Xylitol + 0.25% citric acid	*Y* = 4.18*X* ^0.732^	0.985	Erythritol + 0.30% malic acid	*Y* = 1.67*X* ^1.04^	0.989
Xylitol + 0.45% citric acid	*Y* = 3.34*X* ^0.801^	0.996	Erythritol + 0.55% malic acid	*Y* = 1.39*X* ^1.08^	0.990
Xylitol + 0.80% citric acid	*Y* = 3.18*X* ^0.811^	0.995	Erythritol + 0.95% malic acid	*Y* = 1.02*X* ^1.16^	0.998
Xylitol + 0.30% malic acid	*Y* = 3.75*X* ^0^.^764^	0.996	Sorbitol + 0.25% citric acid	*Y* = 2.24*X* ^0.895^	0.991
Xylitol + 0.55% malic acid	*Y* = 3.12*X* ^0.812^	0.993	Sorbitol + 0.45% citric acid	*Y* = 1.72*X* ^0.984^	0.990
Xylitol + 0.95% malic acid	*Y* = 2.41*X* ^0^.^902^	0.991	Sorbitol + 0.80% citric acid	*Y* = 1.03*X* ^1.12^	0.998
Erythritol + 0.25% citric acid	*Y* = 1.89*X* ^0^.^997^	0.989	Sorbitol + 0.30% malic acid	*Y* = 1.84*X* ^0.961^	0.987
Erythritol + 0.45% citric acid	*Y* = 1.43*X* ^1.08^	0.997	Sorbitol + 0.55% malic acid	*Y* = 1.48*X* ^1^.^02^	0.992
Erythritol + 0.80% citric acid	*Y* = 1.01*X* ^1.19^	0.998	Sorbitol + 0.95% malic acid	*Y* = 0.825*X* ^1.19^	0.992

The results revealed a systematic suppressive effect of acidulants on sweetness perception. For both xylitol and erythritol, across their tested concentration range (0.5% to 15%), the presence of either citric acid or malic acid reduced the perceived sweetness intensity. This suppressive effect was concentration‐dependent, becoming more pronounced as the concentration of the acidulant increased. However, this effect exhibited a saturation point; when the acidulant concentration reached a sufficiently high level, no further increase in sweetness suppression was observed for xylitol and erythritol.

In contrast, the sweetness suppression pattern for sorbitol differed. As illustrated in Figure 3e,f, both acids suppressed the perceived sweetness of sorbitol at low sugar alcohol concentrations (0.5% to 4%). Interestingly, within the medium to high concentration ranges of sorbitol, the suppressive effect of low and moderate acid concentrations was attenuated or diminished. Only high concentrations of either citric acid or malic acid maintained a substantial suppressive effect on sorbitol's sweetness in this higher concentration range.

The systematic suppression of xylitol and erythritol sweetness by acidulants reflected classic taste interaction, where sourness perceptually masked sweetness. The concentration‐dependent effect and its subsequent saturation suggested a perceptual or neural inhibition mechanism with an inherent ceiling, rather than a purely chemical phenomenon. Sorbitol's distinct response, where suppression weakened at medium‐to‐high concentrations except under high acidity, implied that sweetness intensity modulated its susceptibility to suppression. This could be explained by the relative balance of perceptual intensities: stronger sweet signals required stronger sour signals to be effectively suppressed. Alternatively, sorbitol may have differed in its temporal perception or receptor engagement, rendering it less vulnerable to interference from weaker acid levels. These findings underscored the compound‐specific nature of taste interactions, which is essential for designing balanced products with sugar alcohols and acidulants.

### 3.3 Sourness Responses of Acidulants Under Sugar Alcohols Backgrounds

**FIGURE 4:**
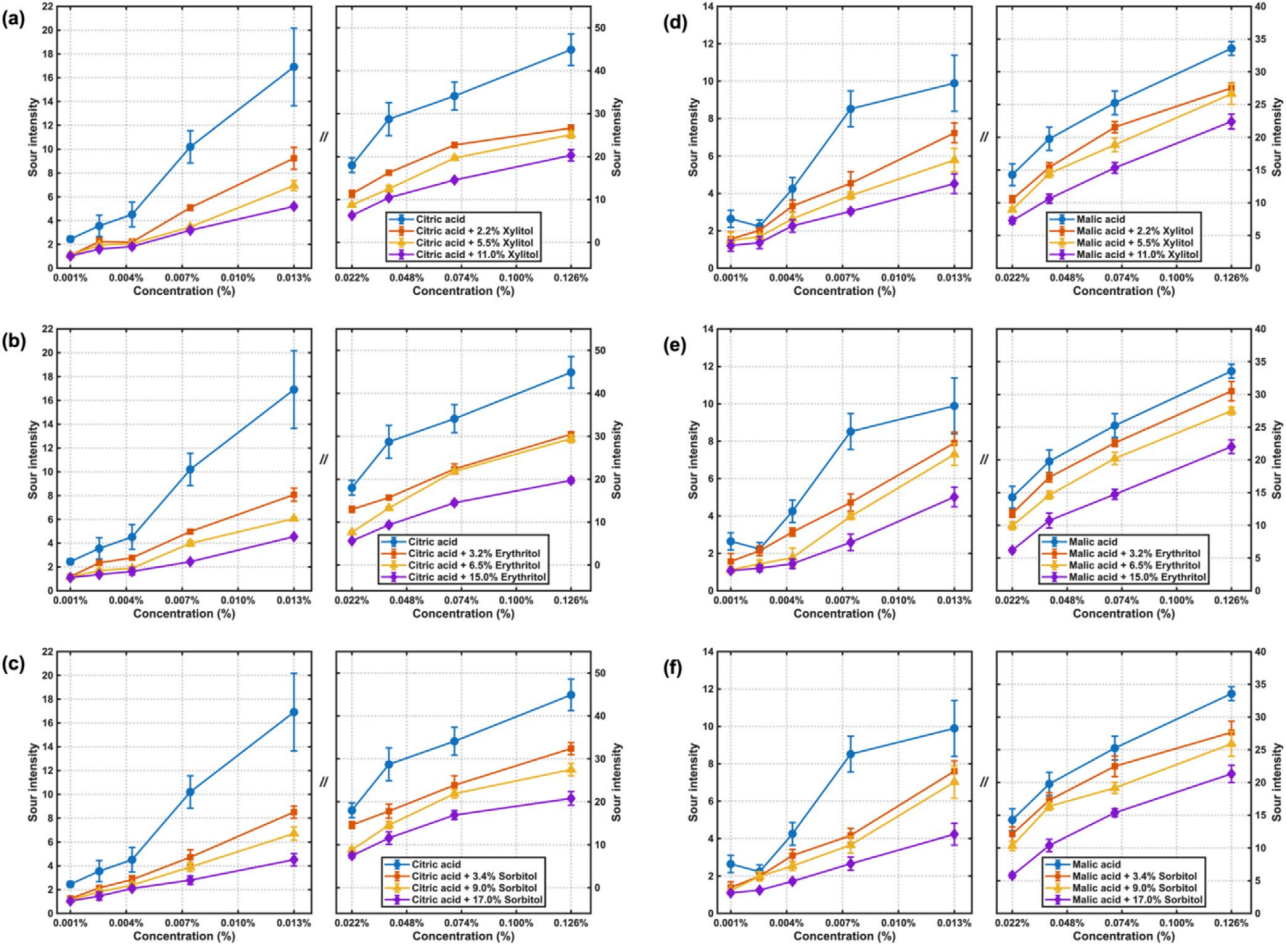
Concentration‐intensity curves of acidulants in liquid sugar alcohols samples: (a) Sourness of citric acid under xylitol; (b) sourness of citric acid under erythritol; (c) sourness of citric acid under sorbitol; (d) sourness of malic acid under xylitol; (e) sourness of malic acid under erythritol; (f) sourness of malic acid under sorbitol.

The perceived sourness intensities of citric acid and malic acid under varying sugar alcohol backgrounds are summarized in Figure 4 which present the psychophysical functions for citric acid across low, moderate, and high concentration backgrounds of xylitol, erythritol, and sorbitol, respectively. The corresponding power‐law fitting parameters and statistical indices are detailed in Table 3.

**TABLE 3:** Fitted equation of interactive response curves of acidulant's sour intensity under sugar alcohols' background.

Substances	Fitted equation	Correlation coefficient (*R* ^2^)	Substances	Fitted equation	Correlation coefficient (*R* ^2^)
Citric acid + 2.2% xylitol	*Y* = 87.16*X* ^0.541^	0.977	Malic acid + 2.2% xylitol	*Y* = 95.16*X* ^0.582^	0.989
Citric acid + 5.5% xylitol	*Y* = 94.42*X* ^0.622^	0.991	Malic acid + 5.5% xylitol	*Y* = 100.13*X* ^0.631^	0.995
Citric acid + 11.0% xylitol	*Y* = 75.93*X* ^0.630^	0.997	Malic acid + 11.0% xylitol	*Y* = 89.71*X* ^0.668^	0.999
Citric acid + 3.2% erythritol	*Y* = 104.82*X* ^0.588^	0.993	Malic acid + 3.2% erythritol	*Y* = 106.13*X* ^0.589^	0.993
Citric acid + 6.5% erythritol	*Y* = 132.58*X* ^0.713^	0.993	Malic acid + 6.5% erythritol	*Y* = 102.72*X* ^0.624^	0.992
Citric acid + 15.0% erythritol	*Y* = 82.01*X* ^0.677^	0.994	Malic acid + 15.0% erythritol	*Y* = 93.03*X* ^0.693^	0.995
Citric acid + 3.4% sorbitol	*Y* = 111.19*X* ^0.583^	0.986	Malic acid + 3.4% sorbitol	*Y* = 93.16*X* ^0.559^	0.981
Citric acid + 9.0% sorbitol	*Y* = 107.35*X* ^0.636^	0.989	Malic acid + 9.0% sorbitol	*Y* = 88.02*X* ^0.574^	0.982
Citric acid + 17.0% sorbitol	*Y* = 79.33*X* ^0.621^	0.986	Malic acid + 17.0% sorbitol	*Y* = 92.17*X* ^0.695^	0.995

For citric acid, the perceived sourness intensity was consistently suppressed by all tested concentrations of the three sugar alcohols. In the low‐concentration range of citric acid, the inhibitory effects from low, moderate, and high concentrations of xylitol, erythritol, and sorbitol were similar in magnitude. As the concentration of citric acid increased, the suppression pattern became more differentiated: the inhibitory effects of xylitol and sorbitol intensified with higher sugar alcohol concentrations. For erythritol, the suppression from its low and moderate concentrations was comparable and weaker than the effect observed at its high concentration.

Figure 4d–f showed the evaluation results for malic acid under the same sugar alcohol backgrounds. A consistent suppressive effect was also observed for malic acid across all sugar alcohol conditions. At low malic acid concentrations, the inhibitory effects from low and moderate sugar alcohol concentrations were comparable for all three sugar alcohols. As the malic acid concentration increased, the suppression of its perceived sourness by all three sugar alcohols intensified progressively with their increasing concentration.

The consistent suppression of sourness by sugar alcohols demonstrated a reciprocal taste interaction, complementing the previously observed sweetness suppression by acidulants. This mutual inhibition suggests the involvement of central cognitive integration mechanisms, where the simultaneous presence of sweet and sour signals leads to a perceptual masking that operates in both directions. The observed concentration‐dependent effects, where higher sugar alcohol levels produced stronger suppression of sourness at elevated acidulant concentrations, indicated that the balance of perceptual intensities played a critical role in determining the magnitude of suppression. The distinct behavior of erythritol in suppressing citric acid sourness, where only its high concentration provided stronger suppression, suggested compound‐specific differences in the temporal dynamics or receptor‐level interactions underlying the suppression effect. These findings further emphasized that taste–taste interactions are bidirectional yet modulated by the specific compounds involved, providing crucial insights for formulating products with balanced sweet–sour profiles.

### 3.4 Interactive Response of Sugar Alcohol—Acidulants Tablet Candy

**FIGURE 5:**
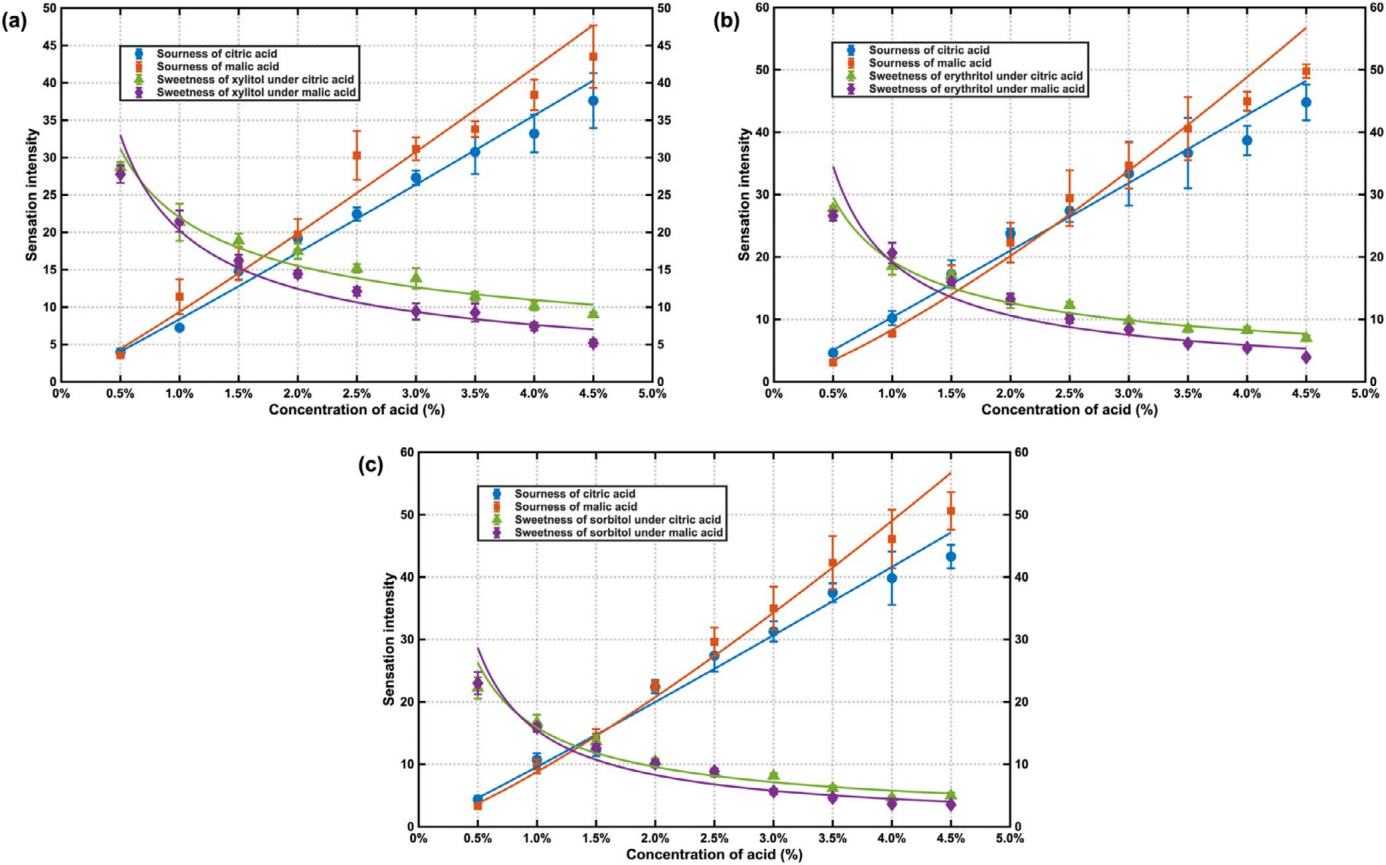
Concentration‐intensity curves of tablet candy samples: (a) Formulated with xylitol and citric acid or malic acid; (b) formulated with erythritol and citric acid or malic acid; (c) formulated with sorbitol and citric acid or malic acid.

The gLMS evaluation results of tablet candies formulated with xylitol, erythritol, and sorbitol (95% matrix) and acidulants were presented in Figure 5a–c. Mutual suppression between the sweetness of sugar alcohols and the sourness of acidulants was consistently observed, as evidenced by characteristic crossover patterns in the perception curves. The fitted equations describing the resultant sweetness and sourness intensities after interaction were summarized in Table 4.

**TABLE 4:** Fitted equation of interactive response curves of sugar alcohol—acidulants tablet candy.

Substances	Perceived intensity	Fitted equation	Correlation coefficient (*R* ^2^)
Xylitol‐citric acid tablet candy	Sweetness	*Y* = 21.99*X* ^−0.502^	0.946
	Sourness	*Y* = 8.38*X* ^1.04^	0.978
Xylitol‐malic acid tablet candy	Sweetness	*Y* = 20.27*X* ^−0.703^	0.904
	Sourness	*Y* = 9.37*X* ^1.08^	0.951
Erythritol‐citric acid tablet candy	Sweetness	*Y* = 19.33*X* ^−0.613^	0.971
	Sourness	*Y* = 10.35*X* ^1.02^	0.971
Erythritol‐malic acid tablet candy	Sweetness	*Y* = 19.15*X* ^−0.851^	0.820
	Sourness	*Y* = 8.35*X* ^1.27^	0.960
Sorbitol‐citric acid tablet candy	Sweetness	*Y* = 15.87*X* ^−0.728^	0.913
	Sourness	*Y* = 9.59*X* ^1.05^	0.976
Sorbitol‐malic acid tablet candy	Sweetness	*Y* = 15.42*X* ^−0.895^	0.869
	Sourness	*Y* = 8.79*X* ^1.24^	0.975

Analysis revealed three key findings: First, the concentration‐perception intensity relationships for all components in the tablet system conformed to Stevens' power law, consistent with observations in liquid systems. Second, the Stevens' exponents for both citric acid and malic acid increased substantially in tablet formulations compared to liquid systems, with an average increase exceeding 79.05%. According to Stevens' power law, the exponent n reflects how rapidly perceived intensity increases with concentration. The > 79.05% average increase in n for acids in tablets indicates a markedly steeper concentration to sourness function in the solid matrix. In practice, this suggests that a lower acid load may be sufficient to achieve a given sourness target in tablets, and that formulation optimization should use smaller dosage steps with tighter control of acid content and dissolution behavior because small variations may yield noticeable sensory differences. This also implies that consumers may experience a sharper increase in sourness with incremental acid addition, potentially shifting perceived balance and acceptance. Third, the sourness perception patterns differed among sugar alcohol matrices. In the xylitol matrix, the Stevens' exponents for citric acid and malic acid were nearly identical (close to 1), indicating comparable sourness perception. However, in both erythritol and sorbitol matrices, the Stevens' exponent for malic acid exceeded that of citric acid by more than 21.26%, suggesting greater panelist sensitivity to concentration changes in malic acid.

The maintained power‐law relationships in tablet candies confirmed the fundamental applicability of Stevens' law to solid food matrices, while the markedly increased exponents for acidulants indicated the alteration of sourness perception dynamics. The observed 79.05% average increase in Stevens' exponents suggested that the tablet microenvironment substantially amplified the perceptual impact of acid concentration variations. This enhancement likely resulted from modified dissolution kinetics and restricted diffusion pathways within the solid matrix, which prolonged oral retention and intensified the temporal concentration gradient of acid molecules at receptor sites.

The matrix‐dependent differentiation between acidulants, particularly the superior sensitivity to malic acid in erythritol and sorbitol matrices, highlighted the role of specific compound interactions in solid formulations. The distinct chemical properties of malic acid, including its dissolution profile and interaction potential with different sugar alcohols, may have facilitated more efficient receptor activation compared to citric acid in these specific matrices. These findings emphasized that sourness perception in solid dosage forms was governed by both physicochemical factors affecting compound release and perceptual factors affecting intensity scaling. From a practical perspective, the heightened sensitivity demanded stricter control of acidulant loading and more precise management of tablet disintegration properties to prevent excessive sourness and ensure consumer acceptance.

### 3.5 Time‐Intensity of Sugar Alcohol—Acidulants Liquid Mixture

**FIGURE 6:**
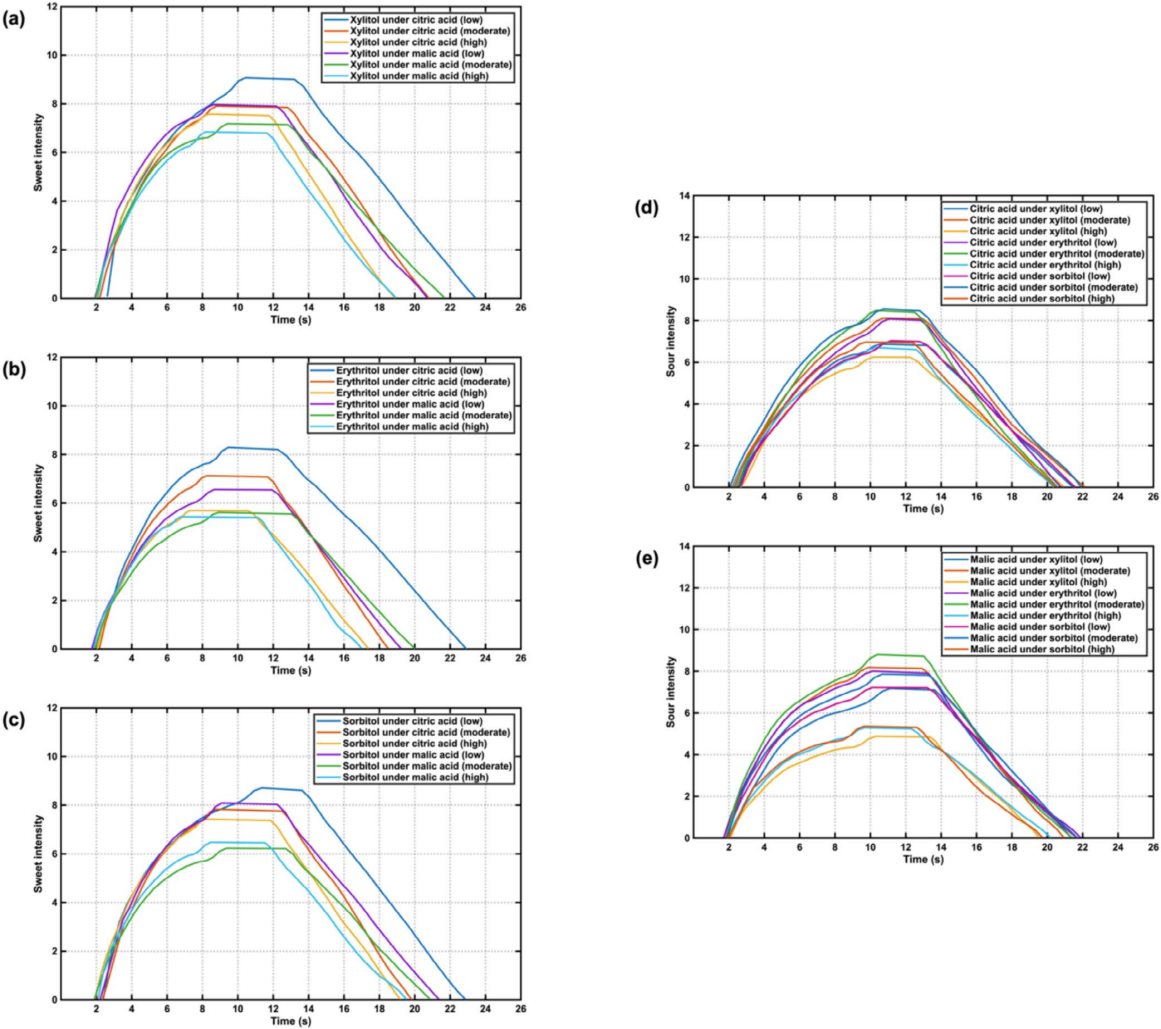
Time‐intensity curves of sugar alcohol‐acidulant mixture liquid samples: (a) Sweetness of xylitol under acidulants; (b) sweetness of erythritol under acidulants; (c) sweetness of sorbitol under acidulants; (d) sourness of citric acid under sugar alcohols; (e) sourness of malic acid under sugar alcohols.

The time‐intensity profiles of perceived sweetness for xylitol, erythritol, and sorbitol in liquid systems under citric acid and malic acid backgrounds were shown in Figure 6a–c, respectively. All sugar alcohols exhibited a consistent trend: sweetness intensity initially increased, reached a plateau, and subsequently declined. The characteristic parameters of the time‐intensity curves were summarized in Tables S3–S5. Based on the Duncan's multiple range test, as citric or malic acid concentration increased, xylitol‐based tablets showed significant decreases in *I*_max_, *T*_start_, *T*_dec_, *T*_end_, and AUC; erythritol‐based tablets showed significant decreases in *I*_max_, Tend, and AUC; and sorbitol‐based tablets showed significant decreases in *I*_max_ and AUC. Across matrices, acid addition therefore produced a monotonic attenuation of sweetness magnitude (*I*_max_, AUC); in xylitol this attenuation also shortened onset, decay, and persistence, yielding a compressed temporal profile. The effect was matrix dependent: xylitol exhibited the strongest temporal compression, erythritol showed reduced magnitude with shorter persistence, and sorbitol exhibited primarily amplitude losses.

The time‐intensity profiles of perceived sourness for citric acid and malic acid in liquid systems under xylitol, erythritol, and sorbitol backgrounds were shown in Figure 6d,e, respectively. Similar to sugar alcohols, all acidulants also exhibited a consistent trend: sourness intensity initially increased, reached a plateau, and subsequently declined. The characteristic parameters of the time‐intensity curves were summarized in Tables S6 and S7. Based on Duncan's multiple range test, as sugar alcohols' concentration increased, the perceived sourness of citric acid and malic acid showed significant decreases in *I*_max_ and AUC.

The bidirectional attenuation observed in the liquid mixtures was consistent with well‐documented mixture suppression between sweetness and sourness: equisweet sweeteners reduced the perceived sourness of citric acid, and reviews of binary taste–taste interactions reported the predominance of sweetness in mixed tastants (Keast and Breslin 2003). In parallel, increases in acid concentration likely increased proton activity in bulk solution and near the epithelium; human psychophysical data indicated that sourness scaled with the combined molar concentrations of hydrogen ions and protonated (undissociated) organic acid species, which together predicted perceived sour intensity with high fidelity (Green et al. 2010). At the same time, higher sugar‐alcohol levels plausibly altered stimulus transport: raising solution viscosity is known to depress taste/flavor intensities, and modeling/experiments showed that added viscosity reduced tastant concentration within papillary structures, providing a transport‐level route to lower magnitude and shorter persistence (Wu and Zhao 2021). Finally, published physicochemical data documented concentration‐dependent viscosity increases in aqueous erythritol, xylitol and sorbitol solutions, offering a matrix‐specific basis for the differences observed across sugar‐alcohol systems (Zhu et al. 2010).

### 3.6 Time‐Intensity of Sugar Alcohol—Acidulants Tablet Candy

**FIGURE 7:**
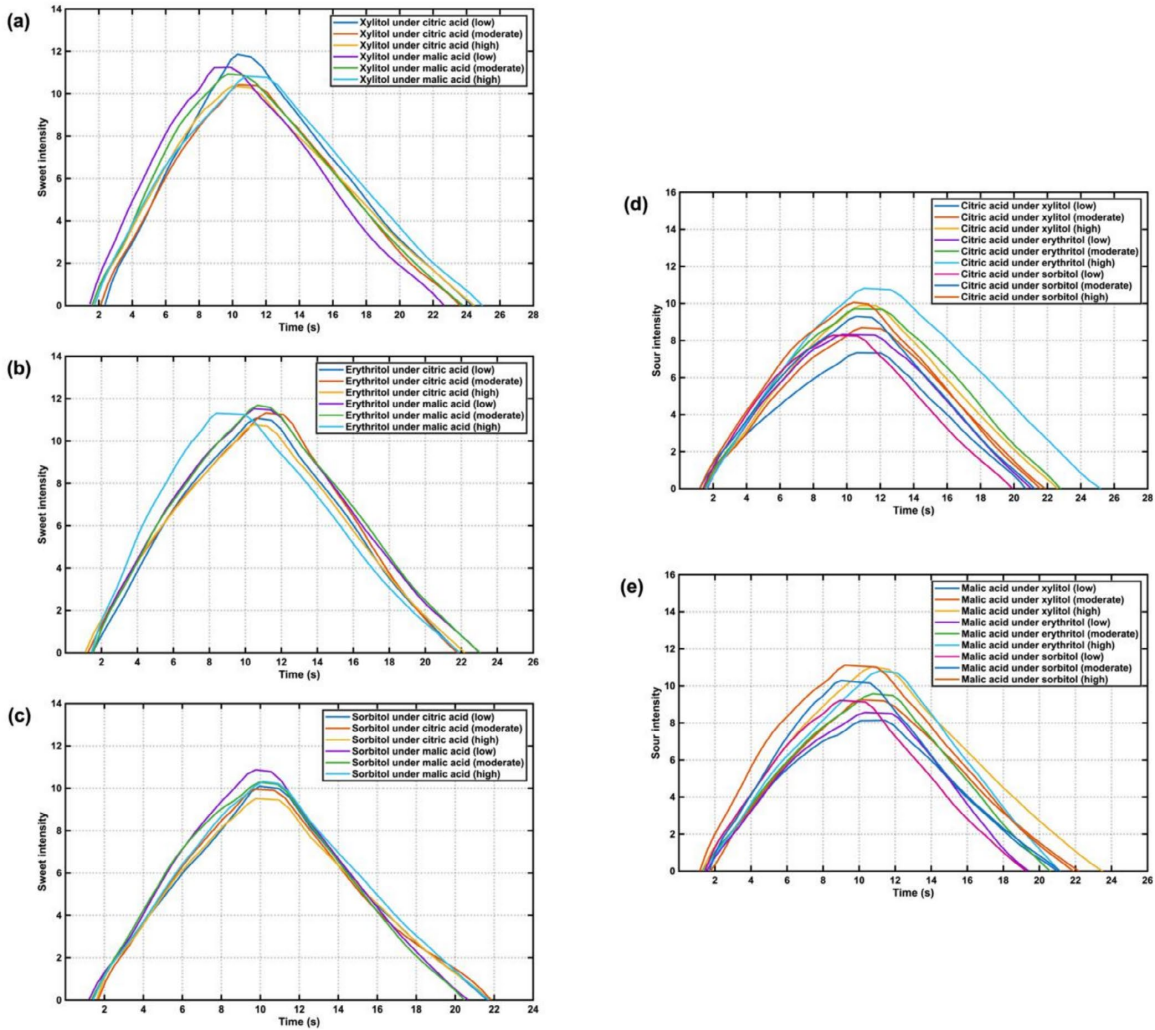
Time‐intensity curves of sugar alcohol‐acidulant mixture tablet candy samples: (a) Sweetness of xylitol under acidulants; (b) sweetness of erythritol under acidulants; (c) sweetness of sorbitol under acidulants; (d) sourness of citric acid under sugar alcohols; (e) sourness of malic acid under sugar alcohols.

The time‐intensity profiles of perceived sweetness for xylitol, erythritol, and sorbitol in tablet candy under citric acid and malic acid backgrounds were shown in Figure 7a–c, respectively. Figure 7d,e showed the perceived sourness for citric acid and malic acid under xylitol, erythritol, and sorbitol backgrounds. Comparative analysis of Figures 6 and 7 revealed that: (1) both sweetness and sourness intensity initially increased, reached a plateau, and subsequently declined; (2) the duration of sweetness and sourness perception in tablet candy samples was comparable to that in the liquid system; (3) the time‐intensity curves of tablet candy samples exhibited steeper profiles, higher peak intensities, and shorter duration at maximum intensity. These results indicated that despite being in a state of sweet–sour taste balance, the dynamic taste perception of tablet candies was more pronounced and transient compared to liquid samples.

The characteristic parameters of the time‐intensity curves were summarized in Tables S8–S12. Based on the Duncan's multiple range test, it could be found that: (1) Significant differences were observed in *I*_max_ and *T*_start_ of xylitol under citric acid and malic acid backgrounds, demonstrating that increasing acidulant concentration suppressed the sweetness intensity and delayed the onset of sweetness perception; (2) Different concentrations of citric acid and malic acid affected the *I*_max_ and *T*_max_ of erythritol, indicating that higher acidulant levels not only reduced the maximum sweetness intensity but also affected the time to reach maximum sweetness; (3) Sorbitol remained largely unaffected by acidulant variations, exhibiting a distinct behavior compared to xylitol and erythritol; (4) Under the 95% sugar alcohol matrix, increasing citric acid content significantly elevated its *I*_max_, *T*_max_, *T*_dec_, and AUC parameters. In contrast, increasing malic acid content only significantly enhanced its *I*_max_ and AUC values. These results suggested that the inhibitory effect of xylitol, erythritol, and sorbitol on sourness perception was weaker against malic acid than against citric acid.

The steeper TI slopes and higher peak intensities observed in tablet candies indicated a more rapid release and intensified perception of tastants in solid matrices compared to liquid systems. This could be attributed to the disintegration process and localized concentration bursts in the oral cavity during tablet dissolution. The comparable total duration of perception suggested that the overall persistence of sweetness and sourness was governed primarily by the physicochemical properties of the sugar alcohols and acidulants, rather than the delivery form.

The compound‐specific effects further elucidated the complexity of taste interactions in solid formulations. The pronounced sensitivity of xylitol and erythritol to acidulant suppression, contrasted with the relative resilience of sorbitol, highlighted the role of molecular structure and dissolution behavior in modulating temporal perception. Moreover, the weaker suppressive effect of all three sugar alcohols against malic acid, as reflected in the sourness TI parameters, suggested that malic acid may engage taste receptors more effectively or exhibit different release kinetics in sugar alcohol matrices. These insights emphasize the importance of considering both dynamic perception and compound‐specific interactions when designing solid confectionery products with desired temporal sensory profiles.

## 4 Conclusions

This study quantified sweet and sour psychophysical functions and their interactions in liquid and tablet systems containing xylitol, erythritol, sorbitol, citric acid, and malic acid. Concentration‐intensity curves followed Stevens' power law above threshold, while subthreshold ratings clustered near the “barely detectable” range. In liquids, bidirectional mixture suppression was evident: acids reduced sweetness and sugar alcohols reduced sourness, with matrix‐dependent effects on amplitude and timing. Tablet candies showed steeper psychophysical scaling for acids than liquids, with more than 79% higher Stevens' exponents, higher peaks, and shorter plateaus, indicating heightened sensitivity and more transient dynamics. Time‐intensity analysis further revealed pronounced temporal compression of sweetness in xylitol, intermediate effects in erythritol, and primarily amplitude losses in sorbitol; malic acid was generally less susceptible to suppression than citric acid.

The insights from this work have strong potential to address a central industry challenge in sugar reduction. By quantifying the relationships among concentration, perceived intensity, and time intensity dynamics, our framework enables more precise prediction and control of taste balance across diverse food and beverage applications. This science guided approach can move product development beyond trial and error by helping manufacturers select ingredient combinations and tune processing or matrix conditions to achieve target sensory profiles, thereby supporting the development of healthier reduced sugar products without sacrificing consumer acceptability.

For practical formulation, the fitted Stevens parameters (*k*, *n*) can be used to translate a desired sweetness or sourness target into a predicted concentration range and to define rational step sizes during optimization. For example, in low sugar candies or beverages, formulators can first estimate the sugar alcohol dose needed to match a sweetness target and then adjust the acidulant level to reach the intended sweet–sour balance while minimizing the risk of overshooting intensity, particularly in tablet matrices where sourness may increase more steeply with concentration.

Several limitations should be acknowledged. This study examined a limited set of acidulants and two matrix types, and we did not directly quantify oral processing factors such as salivary dilution, mastication, or in mouth dissolution dynamics. Future work will extend the framework to additional acidulants and broader sweet–sour systems and will integrate oral processing measurements with mechanistic dissolution or receptor level models to improve prediction across products and consumption contexts.

## Author Contributions

**Chenchen Liu:** writing – original draft, methodology, formal analysis, data curation, software and funding acquisition. **Yuting Wang:** methodology, formal analysis, data curation and software. **Jinxia Bai:** visualization, investigation. **Yuezhong Mao:** supervision, writing – review and editing, conceptualization, funding acquisition and project administration. **Shiyi Tian:** funding acquisition, supervision, writing – review and editing, conceptualization and project administration.

## Funding

This work was supported by China Scholarship Council Program (Grant 202408330436), the Zhejiang Provincial Natural Science Foundation of China (Grant LTGN23C200006), and the “Pioneer” and “Leading Goose” R&D Program of Zhejiang (Grant 2025C04011).

## Ethics Statement

The whole study was approved by the Ethics Committee of Zhejiang Gongshang University (No. 23134703). All participants were asked to provide informed consent prior to participation, with explicit information about data confidentiality and voluntary withdrawal rights.

## Conflicts of Interest

The authors declare no conflicts of interest.

## Supporting information


**TABLE S1:** Proportion of sugar alcohol sweeteners and acidulants in tablet candy for gLMS.
**TABLE S2:** Proportion of sugar alcohol sweeteners and acidulants in tablet candy for TI.
**TABLE S3:** Characteristic parameters of the time‐intensity profiles for xylitol sweetness under liquid conditions across different acidulants.
**TABLE S4:** Characteristic parameters of the time‐intensity profiles for erythritol sweetness under liquid conditions across different acidulants.
**TABLE S5:** Characteristic parameters of the time‐intensity profiles for sorbitol sweetness under liquid conditions across different acidulants.
**TABLE S6:** Characteristic parameters of the time‐intensity profiles for citric acid sourness under liquid conditions across different sugar alcohols.
**TABLE S7:** Characteristic parameters of the time‐intensity profiles for malic acid sourness under liquid conditions across different sugar alcohols.
**TABLE S8:** Characteristic parameters of the time‐intensity profiles for xylitol sweetness under tablet conditions across different acidulants.
**TABLE S9:** Characteristic parameters of the time‐intensity profiles for erythritol sweetness under tablet conditions across different acidulants.
**TABLE S10:** Characteristic parameters of the time‐intensity profiles for sorbitol sweetness under tablet conditions across different acidulants.
**TABLE S11:** Characteristic parameters of the time‐intensity profiles for citric acid sourness under tablet conditions across different sugar alcohols.
**TABLE S12:** Characteristic parameters of the time‐intensity profiles for malic acid sourness under tablet conditions across different sugar alcohols.

## Data Availability

The data that support the findings of this study are available from the corresponding author upon reasonable request.
