# Supplementary material for: Matrix‐Dependent Sweet–Sour Psychophysics: gLMS and Time‐Intensity of Sugar Alcohol‐Acid Mixture in Liquids and Tablets
Source: Food Sci Nutr. 2026 Mar 11;14(3):e71610. doi: 10.1002/fsn3.71610 (PMC12976802; doi:10.1002/fsn3.71610)
Supplement: Supplementary file 1 — TABLE S1: Proportion of sugar alcohol sweeteners and acidulants in tablet candy for gLMS. TABLE S2: Proportion of sugar alcohol sweeteners and acidulants in tablet candy for TI. TABLE S3: Characteristic parameters of the time‐intensity profiles for xylitol sweetness under liquid conditions across different acidulants. TABLE S4: Characteristic parameters of the time‐intensity profiles for erythritol sweetness under liquid conditions across different acidulants. TABLE S5: Characteristic parameters of the time‐intensity profiles for sorbitol sweetness under liquid conditions across different acidulants. TABLE S6: Characteristic parameters of the time‐intensity profiles for citric acid sourness under liquid conditions across different sugar alcohols. TABLE S7: Characteristic parameters of the time‐intensity profiles for malic acid sourness under liquid conditions across different sugar alcohols. TABLE S8: Characteristic parameters of the time‐intensity profiles for xylitol sweetness under tablet conditions across different acidulants. TABLE S9: Characteristic parameters of the time‐intensity profiles for erythritol sweetness under tablet conditions across different acidulants. TABLE S10: Characteristic parameters of the time‐intensity profiles for sorbitol sweetness under tablet conditions across different acidulants. TABLE S11: Characteristic parameters of the time‐intensity profiles for citric acid sourness under tablet conditions across different sugar alcohols. TABLE S12: Characteristic parameters of the time‐intensity profiles for malic acid sourness under tablet conditions across different sugar alcohols. [file FSN3-14-e71610-s001.docx]

Supplementary Table 1 Proportion of sugar alcohol sweeteners and acidulants in tablet candy for gLMS

| Concentration of sugar alcohol, % | Concentration of acidulants, % | Concentration of magnesium stearate, % |
| --- | --- | --- |
| 95% | 0.5 | 4.5 |
|  | 1.0 | 4.0 |
|  | 1.5 | 3.5 |
|  | 2.0 | 3.0 |
|  | 2.5 | 2.5 |
|  | 3.0 | 2.0 |
|  | 3.5 | 1.5 |
|  | 4.0 | 1.0 |
|  | 4.5 | 0.5 |

Supplementary Table 2 Proportion of sugar alcohol sweeteners and acidulants in tablet candy for TI

| Concentration of sugar alcohol, % | Concentration of citric acid, % | | | | Concentration of malic acid, % | | |
| --- | --- | --- | --- | --- | --- | --- | --- |
|  | slightly sour | balanced sweet-sour | predominantly sweet | slightly sour | | balanced sweet-sour | predominantly sweet |
| Xylitol, 95% | 3.07% | 2.50% | 2.04% | 3.08% | | 2.50% | 2.03% |
| Erythritol, 95% | 2.45% | 2.00% | 1.63% | 2.46% | | 2.00% | 1.62% |
| Sorbitol, 95% | 2.45% | 2.00% | 1.63% | 2.46% | | 2.00% | 1.62% |

Supplementary Table 3 Characteristic parameters of the time-intensity profiles for xylitol sweetness under liquid conditions across different acidulants

| Acidulants | I_max_^*^ | T_max_ | T_start_^*^ | T_dec_^*^ | T_end_^*^ | AUC^*^ |
| --- | --- | --- | --- | --- | --- | --- |
| 0.025% Citric acid | 9.04^a^ | 9.72^a^ | 2.75^b^ | 13.85^b^ | 23.96^c^ | 132.25^a^ |
| 0.045% Citric acid | 7.85^b^ | 9.18^a^ | 2.36^ab^ | 13.26^ab^ | 21.14^ab^ | 98.98^b^ |
| 0.080% Citric acid | 7.54^b^ | 8.38^a^ | 2.10^a^ | 12.15^a^ | 19.20^a^ | 85.92^b^ |
| 0.030% Malic acid | 7.96^b^ | 8.76^a^ | 2.05^a^ | 13.38^ab^ | 21.74^b^ | 100.44^b^ |
| 0.055% Malic acid | 7.16^b^ | 8.69^a^ | 2.04^a^ | 12.70^ab^ | 21.14^ab^ | 93.38^b^ |
| 0.095% Malic acid | 7.08^b^ | 8.20^a^ | 2.34^ab^ | 12.03^a^ | 19.14^a^ | 77.67^b^ |

* Indicated statistically significant differences in the sample characteristic values by Duncan’s multiple range test.

Supplementary Table 4 Characteristic parameters of the time-intensity profiles for erythritol sweetness under liquid conditions across different acidulants

| Acidulants | I_max_^*^ | T_max_ | T_start_^*^ | T_dec_^*^ | T_end_^*^ | AUC^*^ |
| --- | --- | --- | --- | --- | --- | --- |
| 0.025% Citric acid | 8.33^d^ | 8.70^ab^ | 2.69^c^ | 12.91^ab^ | 23.54^d^ | 120.22^c^ |
| 0.045% Citric acid | 7.12^c^ | 8.35^ab^ | 2.28^bc^ | 12.06^ab^ | 18.72^abc^ | 78.03^b^ |
| 0.080% Citric acid | 5.69^ab^ | 7.52^ab^ | 2.15^ab^ | 11.53^a^ | 17.68^ab^ | 60.12^ab^ |
| 0.030% Malic acid | 6.62^bc^ | 9.22^b^ | 2.10^ab^ | 13.37^b^ | 20.46^c^ | 79.24^b^ |
| 0.055% Malic acid | 5.84^ab^ | 8.30^ab^ | 1.81^a^ | 12.38^ab^ | 19.59^bc^ | 74.41^ab^ |
| 0.095% Malic acid | 5.56^a^ | 7.23^a^ | 2.00^ab^ | 11.60^a^ | 17.19^a^ | 56.93^a^ |

* Indicated statistically significant differences in the sample characteristic values by Duncan’s multiple range test.

Supplementary Table 5 Characteristic parameters of the time-intensity profiles for sorbitol sweetness under liquid conditions across different acidulants

| Acidulants | I_max_^*^ | T_max_ | T_start_ | T_dec_ | T_end_^*^ | AUC^*^ |
| --- | --- | --- | --- | --- | --- | --- |
| 0.025% Citric acid | 8.56^c^ | 9.91^b^ | 2.55^a^ | 14.18^b^ | 23.45^b^ | 128.31^d^ |
| 0.045% Citric acid | 7.71^bc^ | 8.75^ab^ | 2.40^a^ | 12.94^a^ | 20.08^a^ | 92.31^bc^ |
| 0.080% Citric acid | 7.31^b^ | 8.49^ab^ | 2.12^a^ | 12.39^a^ | 19.63^a^ | 86.57^abc^ |
| 0.030% Malic acid | 8.10^bc^ | 9.00^ab^ | 2.10^a^ | 12.91^a^ | 20.95^a^ | 102.04^c^ |
| 0.055% Malic acid | 6.21^a^ | 8.87^ab^ | 2.34^a^ | 12.67^a^ | 21.80^ab^ | 77.74^ab^ |
| 0.095% Malic acid | 6.24^a^ | 7.84^a^ | 2.35^a^ | 12.00^a^ | 19.53^a^ | 69.06^a^ |

* Indicated statistically significant differences in the sample characteristic values by Duncan’s multiple range test.

Supplementary Table 6 Characteristic parameters of the time-intensity profiles for citric acid sourness under liquid conditions across different sugar alcohols

| Sugar alcohols | I_max_^*^ | T_max_ | T_start_ | T_dec_ | T_end_ | AUC^*^ |
| --- | --- | --- | --- | --- | --- | --- |
| 2.2% Xylitol | 8.00^b^ | 10.27^a^ | 2.33^ab^ | 13.53^a^ | 22.66^a^ | 103.56^bc^ |
| 5.5% Xylitol | 6.97^a^ | 10.04^a^ | 2.66^ab^ | 13.76^a^ | 21.82^a^ | 84.58^ab^ |
| 11.0% Xylitol | 6.23^a^ | 9.26^a^ | 2.70^ab^ | 13.40^a^ | 21.28^a^ | 74.75^a^ |
| 3.2% Erythritol | 8.24^b^ | 10.26^a^ | 2.85^b^ | 13.58^a^ | 21.24^a^ | 95.05^abc^ |
| 6.5% Erythritol | 7.89^b^ | 9.75^a^ | 2.69^ab^ | 13.01^a^ | 20.94^a^ | 94.89^abc^ |
| 15.0% Erythritol | 6.67^a^ | 9.66^a^ | 2.37^ab^ | 13.14^a^ | 21.15^a^ | 80.05^a^ |
| 3.4% Sorbitol | 8.44^b^ | 10.30^a^ | 2.76^ab^ | 14.11^a^ | 22.51^a^ | 109.98^c^ |
| 9.0% Sorbitol | 6.96^a^ | 9.85^a^ | 2.18^a^ | 13.27^a^ | 21.98^a^ | 85.80^ab^ |
| 17.0% Sorbitol | 6.86^a^ | 9.24^a^ | 2.47^ab^ | 13.37^a^ | 20.95^a^ | 82.36^ab^ |

* Indicated statistically significant differences in the sample characteristic values by Duncan’s multiple range test.

Supplementary Table 7 Characteristic parameters of the time-intensity profiles for malic acid sourness under liquid conditions across different sugar alcohols

| Sugar alcohols | I_max_^*^ | T_max_ | T_start_ | T_dec_ | T_end_ | AUC^*^ |
| --- | --- | --- | --- | --- | --- | --- |
| 2.2% Xylitol | 8.00^bc^ | 10.43^a^ | 2.27^a^ | 14.08^a^ | 21.72^a^ | 103.22^bc^ |
| 5.5% Xylitol | 7.12^b^ | 9.24^a^ | 1.98^a^ | 13.38^a^ | 21.30^a^ | 90.91^b^ |
| 11.0% Xylitol | 5.11^a^ | 10.05^a^ | 2.73^a^ | 13.91^a^ | 20.03^a^ | 60.16^a^ |
| 3.2% Erythritol | 8.58^c^ | 9.86^a^ | 1.84^a^ | 13.80^a^ | 22.51^a^ | 111.54^c^ |
| 6.5% Erythritol | 7.90^bc^ | 9.41^a^ | 1.98^a^ | 13.52^a^ | 21.79^a^ | 107.91^bc^ |
| 15.0% Erythritol | 5.34^a^ | 9.23^a^ | 2.05^a^ | 13.39^a^ | 20.79^a^ | 65.34^a^ |
| 3.4% Sorbitol | 7.83^bc^ | 9.97^a^ | 2.08^a^ | 13.68^a^ | 22.14^a^ | 102.45^bc^ |
| 9.0% Sorbitol | 7.31^b^ | 9.33^a^ | 2.09^a^ | 13.48^a^ | 22.08^a^ | 97.76^bc^ |
| 17.0% Sorbitol | 5.53^a^ | 9.11^a^ | 1.90^a^ | 13.02^a^ | 20.11^a^ | 65.55^a^ |

* Indicated statistically significant differences in the sample characteristic values by Duncan’s multiple range test.

Supplementary Table 8 Characteristic parameters of the time-intensity profiles for xylitol sweetness under tablet conditions across different acidulants

| Acidulants | I_max_^*^ | T_max_ | T_start_^*^ | T_dec_ | T_end_ | AUC |
| --- | --- | --- | --- | --- | --- | --- |
| 2.04% Citric acid | 11.15^c^ | 10.03^ab^ | 2.52^c^ | 12.88^ab^ | 25.05^a^ | 140.66^a^ |
| 2.50% Citric acid | 10.00^ab^ | 9.64^ab^ | 2.24^bc^ | 12.45^ab^ | 24.28^a^ | 131.59^a^ |
| 3.07% Citric acid | 9.78^a^ | 9.34^ab^ | 1.94^ab^ | 12.36^ab^ | 25.24^a^ | 126.85^a^ |
| 2.03% Malic acid | 10.73^bc^ | 8.24^a^ | 1.67^a^ | 11.29^a^ | 22.65^a^ | 135.29^a^ |
| 2.50% Malic acid | 10.36^ab^ | 9.00^ab^ | 1.84^ab^ | 11.85^ab^ | 24.90^a^ | 135.31^a^ |
| 3.08% Malic acid | 10.37^ab^ | 10.39^b^ | 1.89^ab^ | 13.27^b^ | 26.32^a^ | 146.06^a^ |

* Indicated statistically significant differences in the sample characteristic values by Duncan’s multiple range test.

Supplementary Table 9 Characteristic parameters of the time-intensity profiles for erythritol sweetness under tablet conditions across different acidulants

| Acidulants | I_max_^*^ | T_max_^*^ | T_start_ | T_dec_ | T_end_ | AUC |
| --- | --- | --- | --- | --- | --- | --- |
| 1.63% Citric acid | 10.56^bc^ | 9.75^ab^ | 1.69^ab^ | 12.50^ab^ | 22.53^a^ | 124.46^a^ |
| 2.00% Citric acid | 10.28^ab^ | 9.67^ab^ | 1.43^ab^ | 13.12^b^ | 22.40^a^ | 130.10^a^ |
| 2.45% Citric acid | 10.04^a^ | 10.44^b^ | 1.25^a^ | 12.57^ab^ | 22.73^a^ | 124.71^a^ |
| 1.62% Malic acid | 10.86^c^ | 9.49^ab^ | 1.77^b^ | 12.55^ab^ | 23.81^a^ | 138.19^a^ |
| 2.00% Malic acid | 10.76^bc^ | 9.42^ab^ | 1.62^ab^ | 12.27^ab^ | 23.73^a^ | 140.84^a^ |
| 2.46% Malic acid | 10.52^bc^ | 8.54^a^ | 1.57^ab^ | 11.28^a^ | 22.61^a^ | 126.85^a^ |

* Indicated statistically significant differences in the sample characteristic values by Duncan’s multiple range test.

Supplementary Table 10 Characteristic parameters of the time-intensity profiles for sorbitol sweetness under tablet conditions across different acidulants

| Acidulants | I_max_ | T_max_ | T_start_ | T_dec_ | T_end_ | AUC |
| --- | --- | --- | --- | --- | --- | --- |
| 1.63% Citric acid | 9.42^ab^ | 9.31^a^ | 1.65^ab^ | 12.17^a^ | 22.53^a^ | 108.92^a^ |
| 2.00% Citric acid | 9.23^ab^ | 8.93^a^ | 1.88^b^ | 11.99^a^ | 22.75^a^ | 108.19^a^ |
| 2.45% Citric acid | 9.02^a^ | 9.26^a^ | 1.80^ab^ | 12.01^a^ | 22.99^a^ | 108.33^a^ |
| 1.62% Malic acid | 10.00^b^ | 8.74^a^ | 1.38^a^ | 11.54^a^ | 21.23^a^ | 111.93^a^ |
| 2.00% Malic acid | 9.64^ab^ | 9.08^a^ | 1.58^ab^ | 11.87^a^ | 21.05^a^ | 109.11^a^ |
| 2.46% Malic acid | 9.62^ab^ | 9.16^a^ | 1.55^ab^ | 11.87^a^ | 22.83^a^ | 116.21^a^ |

* Indicated statistically significant differences in the sample characteristic values by Duncan’s multiple range test.

Supplementary Table 11 Characteristic parameters of the time-intensity profiles for citric acid sourness under tablet conditions across different sugar alcohols

| Samples | I_max_^*^ | T_max_^*^ | T_start_ | T_dec_^*^ | T_end_ | AUC^*^ |
| --- | --- | --- | --- | --- | --- | --- |
| 95% Xylitol+2.04% Citric acid | 7.01^a^ | 9.65^bc^ | 1.58^a^ | 12.93^b^ | 20.83^a^ | 86.80^a^ |
| 95% Xylitol +2.50% Citric acid | 8.36^b^ | 10.10^bc^ | 1.79^a^ | 13.07^b^ | 22.04^ab^ | 109.04^ab^ |
| 95% Xylitol +3.07% Citric acid | 9.36^c^ | 9.97^bc^ | 1.87^a^ | 12.95^b^ | 23.53^ab^ | 122.13^abc^ |
| 95% Erythritol+1.63% Citric acid | 8.31^b^ | 9.05^ab^ | 1.87^a^ | 12.18^ab^ | 21.69^ab^ | 103.97^ab^ |
| 95% Erythritol +2.00% Citric acid | 9.41^c^ | 9.55^bc^ | 1.71^a^ | 12.82^b^ | 21.89^ab^ | 131.44^bc^ |
| 95% Erythritol +2.45% Citric acid | 10.51^d^ | 10.48^d^ | 1.91^a^ | 13.22^b^ | 26.17^b^ | 157.63^c^ |
| 95% Sorbitol+1.63% Citric acid | 8.05^b^ | 8.27a | 1.55^a^ | 11.20^a^ | 20.73^a^ | 93.43^a^ |
| 95% Sorbitol +2.00% Citric acid | 8.75^bc^ | 9.17^ab^ | 1.50^a^ | 12.15^ab^ | 22.06^ab^ | 108.33^ab^ |
| 95% Sorbitol +2.45% Citric acid | 9.56^c^ | 9.2^ab^ | 1.34^a^ | 12.04^ab^ | 22.48^ab^ | 122.54^abc^ |

* Indicated statistically significant differences in the sample characteristic values by Duncan’s multiple range test.

Supplementary Table 12 Characteristic parameters of the time-intensity profiles for malic acid sourness under tablet conditions across different sugar alcohols

| Samples | I_max_^*^ | T_max_ | T_start_ | T_dec_ | T_end_ | AUC^*^ |
| --- | --- | --- | --- | --- | --- | --- |
| 95% Xylitol+2.03% Malic acid | 7.92^a^ | 9.48^ab^ | 1.87^a^ | 12.37^ab^ | 21.63^ab^ | 92.54^a^ |
| 95% Xylitol +2.50% Malic acid | 9.01^bc^ | 9.33^ab^ | 1.99^a^ | 12.24^ab^ | 22.90^ab^ | 110.60^ab^ |
| 95% Xylitol +3.08% Malic acid | 10.32^de^ | 9.56^ab^ | 1.73^a^ | 12.48^ab^ | 24.32^b^ | 135.69^c^ |
| 95% Erythritol+1.62% Malic acid | 8.22^a^ | 9.81^b^ | 1.88^a^ | 12.67^ab^ | 20.08^a^ | 92.26^a^ |
| 95% Erythritol +2.00% Malic acid | 9.10^bc^ | 9.95^b^ | 1.57^a^ | 12.76^ab^ | 21.18^ab^ | 106.60^ab^ |
| 95% Erythritol +2.46% Malic acid | 10.11^de^ | 10.29^b^ | 1.68^a^ | 13.10^b^ | 21.85^ab^ | 120.56^bc^ |
| 95% Sorbitol+1.62% Malic acid | 8.67^ab^ | 8.03^a^ | 1.75^a^ | 11.20^a^ | 20.08^a^ | 94.34^a^ |
| 95% Sorbitol +2.00% Malic acid | 9.72^cd^ | 8.52^ab^ | 1.86^a^ | 11.40^ab^ | 21.07^ab^ | 107.74^ab^ |
| 95% Sorbitol +2.46% Malic acid | 10.71^e^ | 8.73^ab^ | 1.40^a^ | 11.56^ab^ | 21.82^ab^ | 130.86^bc^ |

* Indicated statistically significant differences in the sample characteristic values by Duncan’s multiple range test.
